# Physical Fitness and Functional Ability of Children with Intellectual Disability: Effects of a Short-Term Daily Treadmill Intervention

**DOI:** 10.1100/tsw.2004.97

**Published:** 2004-06-14

**Authors:** Meir Lotan, Eli Isakov, Shlomo Kessel, Joav Merrick

**Affiliations:** Zvi Quittman Residential Center, The Millie Shime Campus, Elwyn, Jerusalem, Israel; ^2^ 2Orthopedic Department, Loewenstein Rehabilitation Hospital, Raanana, Sackler School of Medicine, Tel Aviv University, Israel; ^3^ Sarah Herzog Children Village, Afula, Israel; ^4^ National Institute of Child Health and Human Development, Office of the Medical Director, Division for Mental Retardation, Ministry of Social Affairs, Jerusalem and Zusman Child Development Center, Division of Pediatrics and Community Health, Ben Gurion University, Beer-Sheva, Israel

**Keywords:** mental retardation, developmental disability, intellectual disability, physical fitness, human development, public health, Israel

## Abstract

Persons with intellectual disability (ID) and associated multiple disabilities have been found by many researchers to be a population with deficient physical fitness measures, which can be explained by an inactive lifestyle, a result of lack of awareness of the positive physical effects of physical exercise, or lack of motivation for any motor activity. Various plans for physical exercise have been put forward, but many are found impractical in nonresearch-based intervention. In this study, 15 children with ID on a motor functioning level of 7—14 months used a treadmill daily for 2 months. Our findings indicated a most significant improvement in the level of physical fitness of the participants (p < 0.005), as measured by pulse at rest and during effort. The improvement in physical fitness modestly (r = 0.5), but significantly (p < 0.05), correlated with a significant (p < 0.0007) improvement in functional ability of the participating children. Further examination a year after intervention terminated showed a return to preintervention pulse-at-rest values. The research examined the treadmill training method and found that it can be operated with the support of an unskilled staff person under the supervision of a physiotherapist. The research was performed under real-life conditions, enabling relatively easy implementation in the existing conditions of special education centers. This method is a type of exercise that is easy to operate without entailing long-term budgetary expenses and might improve the health status of children with ID, who are a population at risk for developing heart-related diseases at a young age.

## INTRODUCTION

Persons with intellectual disability (ID) have been found by many researchers to lack in physical fitness when compared to peers without ID[1,2,3,4,5,6,7,8,9]. Fernhall et al.[10] went so far as to state that “the physical fitness is so poor that cognitively disabled 20- to 30-year-old men present a cardiovascular level appropriate for people without cognitive disorders, who are 30–40 years older, or men who had a heart attack”.

There can be many reasons and explanations for the low levels of fitness, like a passive lifestyle[7,8,11], low motivation[12], psychological or physiological barriers[3], or motor passivity[3]. Of all the factors examined, it was found that “inactive life style was the most harmful to physical fitness”[13]. The lack of physical fitness can lead to early aging phenomena and states of illness compared with the population with no cognitive disorders[14]. Vaccaro and Mahon[15] suggested that: “it has been proved today that heart illnesses are childhood illnesses, although the clinical signs of these illnesses are only observed later on in life… and therefore it is necessary to locate the children at-risk for these illnesses… and initiate intervention programs… (which will also include)… increased physical activity. This activity (and others)… was found to have the ability to change the risk factors for heart illnesses.”

In light of these findings, several intervention programs were implemented over the years[4,16,17,18] with some using the treadmill[7]. These programs achieved improvement in various fields, such as muscle tolerance[17], physical fitness and lower pulse per minute[18], and muscle power[11]. The treadmill has served as an instrument for examination and exercise in medicine from the 1950s[19], but only few studies performed with the population of persons with ID.

The purpose of this study was to investigate if it would be possible to use the treadmill with severely disabled children in a daily exercise program in order to improve their physical fitness, to investigate the relation between physical fitness achievements and functional abilities, and to discover if the exercise could be performed by unskilled staff members to ensure low costs.

## METHODS

The research population consisted of 15 children aged 5–10 years (mean: 7.9; 7 girls and 8 boys) attending the educational facility Beit Issie Shapiro, Raanana, Israel. All the children presented with motor ability in the range of 7- to 15-month-old infants and low muscle tone. Of the group, 4 children had moderate ID, 8 severe, and 3 profound.

Each guardian approved the study and the physician in charge gave informed consent. The local review board gave approval as well.

The research model compared the physiological measures (functional examinations, examination in walking laboratory, and examination of pulse during rest and effort) about 2 months before operating the therapeutic intervention, on beginning the therapeutic intervention (a daily exercise of 20–30 min on the treadmill), and at the end of the intervention (2 months after intervention initiation). The large number of interfering factors did not enable compatibility among a similar population. Thus, using the group as its own control neutralized interfering factors, such as age, diagnosis, functional level, behavior problems, quantity, type, and quality of paramedical treatments.

In order to examine whether this intervention was realistic and may be performed without interfering with the educational schedules of the children involved, exercise sessions were coordinated with the teachers.

The exercise instrument was a Trimline treadmill, model 1400. Pulse was measured during rest and continuously during exercise by a CBI, model 3301, a pulse and oxygen saturation measurer. The children participating in the research exercised daily for the 2 months of the research for an average of 19.9 min every day. The children began walking at an average initial speed of 1.7 kph and ended at an average speed of 2.7 kph. The average duration of each exercise session was 6.7 min at the beginning of the experimental intervention and 28.8 min at the end of the experimental intervention. The average capacity during the first session was 223 kcal (kilocalories) and the average capacity during the final session was 1,965 kcal. (The capacity was calculated by the formula: child weight × duration of exercise in minutes × walking speed × cosine of the instrument's angle of elevation.)

The treatment program for each child (speed, duration, instrument elevation) was determined in advance by the physiotherapist. The trainer was responsible for placing the children on the exercise machine, performing the predetermined program, and registering the maximal pulse at the end of the session. During the months of exercise, the children exercised 37.7 days on average (756.6 exercise minutes), with an average capacity of 1,217 kcal. The statistical calculations were performed on the differences between the examinations in a two-tailed paired student t-test and the correlation between changes in physical fitness and functional ability was performed by Pearson's r.

Heart rate at rest and during exercise was measured on three occasions ($O_1$, $O_2$, $O_3$). Functional ability was measured at three different occasions ($O_1$, $O_2$, $O_3$) using a tool that was constructed especially for the present intervention, due to lack of an appropriate tool to measure the change in functional ability of cognitively impaired children during a short-term intervention. The tool used increments holding face validity such as time (minutes) and distance (meters). The tool used has been supervised and corrected prior to its use by three experienced physical therapists (authors not included) not related with the present intervention, thus giving the tool content validity. 0.95 Inter-rater reliability was found on 15% of the measurements; 0.8 reproducibility value was found between baseline and second ($O_1$-$O_2$) measurements.

## RESULTS

Pulse at rest and pulse during activity were measured as indicators of the degree of improvement in physical fitness. A significant improvement was noted on both indices. Functional ability was measured three times adjacent to pulse measurements and found significantly improved. At program termination, improvement in pulse and functional ability were found to correlate. A year after program termination, the participants' heart rates were elevated almost to preintervention values.

Data on pulse at rest from the first examination ($O_1$ = 2 months before beginning the exercise plan) was subtracted from data on pulse at rest from the second examination (before beginning the exercise sessions = $O_2$), and data on pulse at rest from second examination (= $O_2$) was subtracted from data of pulse at rest from the third examination (at the end of the exercise program = $O_3$). Two-tailed paired student t-test was performed on both of these groups of numbers and showed a significant improvement in the pulse-at-rest differences (Bilateral, df = 14, $p < 0.016$).

The differences in the pulse at rest between the first ($O_1$) and second examinations ($O_2$) were not constant as some of the children registered an increase in pulse measured, while others registered a decrease. The average change was 0.5. In other words, if there was any change at all, it was minute (upward directed). Between the second ($O_2$) and third examinations ($O_3$, the period of therapeutic intervention), most of the measurements indicated a lower pulse and the average decrease in pulse per child was -13.5.

A comparison of the average pulse-at-rest measurements from first to third examination of all subjects (see Fig. 1) indicated clear decrease in examination $O_3$, in relation to the two prior examinations.

Data on pulse during activity was performed in an identical manner to the processing of the pulse-at-rest data. The data of pulse during effort from the first examination ($O_1$ = 2 months before beginning the exercise plan) was subtracted from data of pulse during effort from the second examination and data of pulse during effort from second examination ($O_2$ = before beginning the exercise sessions) was subtracted from data of pulse during effort from the third examination ($O_3$ = at the end of the exercise sessions). The statistical calculation performed on both of these groups of numbers (the differences between examinations 1 and 2, and 2 to 3) was a two-tailed paired student t-test and a significant improvement was found in the pulse during effort differences (bilateral, df = 14, $p < 0.000$).

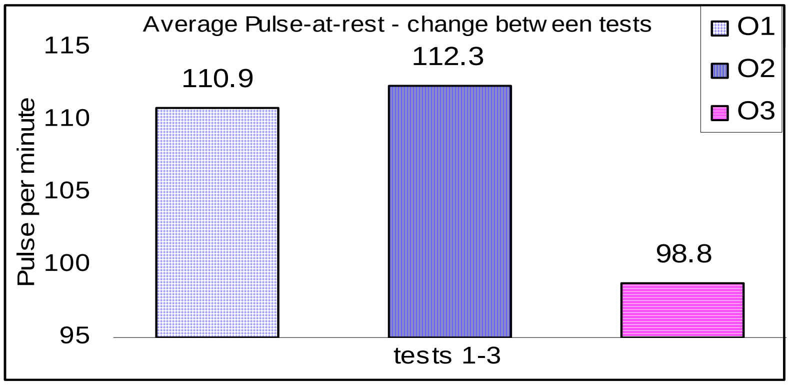

**FIGURE 1**. Change in average pulse at rest across tests. The figure represents the average heart-at-rest scores for all participants (n = 15) at tests $O_1$ (2 months before program initiation), $O_2$ (at the beginning of the intervention program), and $O_3$ (at the end of the 2-month intervention program).

The average of the pulse change during the first period ($O_1$-$O_2$) was 0.67; in other words, there was actually no change. This was in contrast to the period of intervention, in which the average change in the pulse during effort was –24.13 per child, a clear and significant pulse drop (see Fig. 2). During the 2 months prior to the therapeutic intervention, no change occurred in the pulse of the research population, while the average pulse per minute during effort dropped significantly and obviously following the intervention.

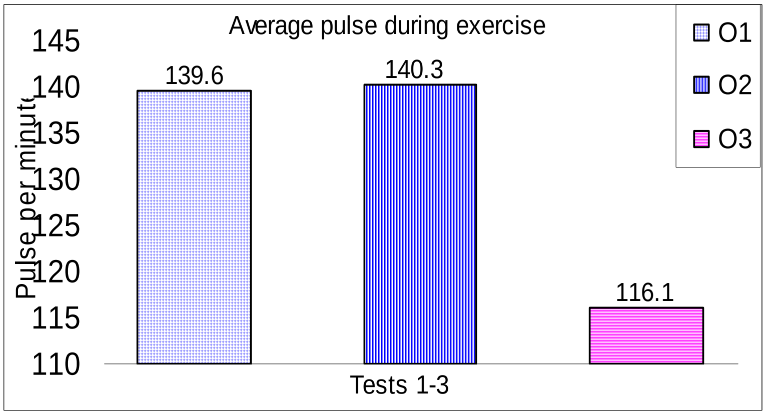

**FIGURE 2**. Change in average pulse during exercise. The figure represents the average heart during exercise scores for all participants (n = 15) at tests $O_1$ (2 months before program initiation), $O_2$ (at the beginning of the intervention program), and $O_3$ (at the end of the 2-month intervention program).

No statistically significant change in functional ability was found in the period prior to intervention ($O_2$-$O_1$) using a two-tailed paired student t-test. On the other hand, a clear and obvious improvement (bilateral, df = 14, $p < 0.0007$) was found between functional tests (see Fig. 3), performed at the beginning and the end of the intervention ($O_3$-$O_2$).

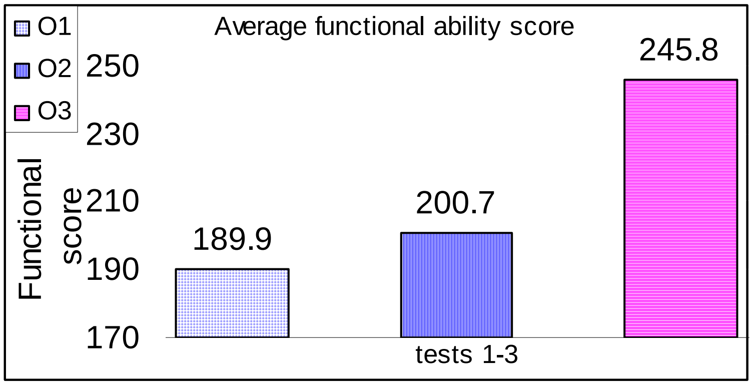

**FIGURE 3.** Average change in functional scores. The figure represents the average functional scores for all participants (n = 15) at tests $O_1$ (2 months before program initiation), $O_2$ (at the beginning of the intervention program), and $O_3$ (at the end of the 2-month intervention program).

A Pearson's r correlation between aerobic improvement (represented by reduction in heart rate at rest) and functional ability (represented by higher scores in the functional test) showed a moderate (r = -49), but significant (Unilateral, df = 14, $p < 0.031$), correlation (see Fig. 4).

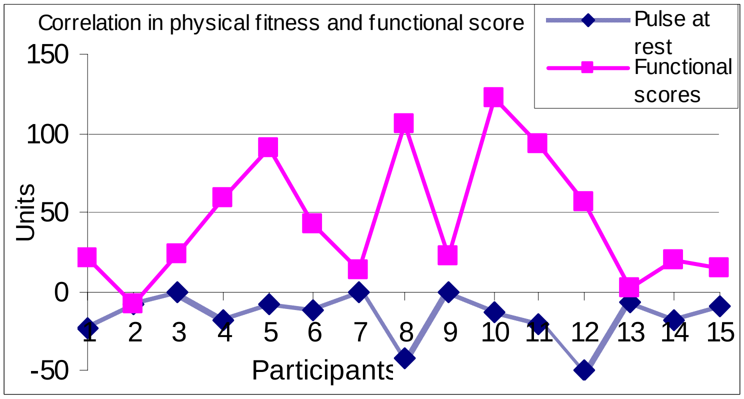

**FIGURE 4**. A correlation between change in pulse at rest and change in functional scores. The figure represents the correlation between the change in individual functional score vs. the change in individual heart rate at rest for each participant between tests $O_2$ (at the beginning of the intervention program) and $O_3$ (at the end of the intervention program).

A further enhancement of the results was established a year after completion of the above-mentioned intervention. A follow-up measurement of heart rate at rest was performed on the children participating in the research (4 children moved to a different facility, hence measurements were performed on the 11 remaining children). The figure presents average heart rate at rest (see Fig. 5); the new test was named $O_4$ (represented by the column with circles). Results were not statistically significant however, probably due to the small number of participants ($O_2$-$O_3$ Bilateral, df = 10, $p < 0.07$; $O_3$-$O_4$ Bilateral, df = 10, $p < 0.26$).

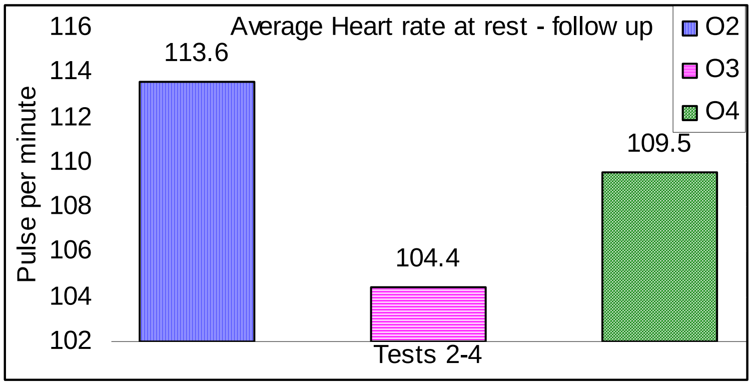

**FIGURE 5**. A change in heart rate a year after intervention terminated. The figure represents the average heart-at-rest scores for all participants still at daycare center (n = 11) at $O_2$ (at the beginning of the intervention program), $O_3$ (at the end of the 2-month intervention program), and $O_4$ (a year after intervention termination).

## DISCUSSION

The current research was performed in order to try and answer a number of key questions related to the treatment of ID children.

In the current research, the children were supervised/trained by a National Service volunteer, thus indicating that it was possible to train unskilled staff to serve as motor operators on the specific level for which they were trained during a short (2 weeks) period of time. The findings are similar to those of previous studies[12]. The execution of such an intervention program enables increasing the treatment intensiveness for cognitively disabled populations at a lower expense than that required by employing paramedical professionals, and can be used as a supplementary program to physical therapy.

This intervention was operated in a special education school, with cooperation between the researcher and the educational staff. This indicated that such a program could be operated in the actual terms of an educational institution without changing the children's educational curriculum. Our study reinforced the appeal from experts on physical exercise for populations with ID[7], who advocated to operate research programs that will not only have positive results for the participants, but also applicable in daily life.

The physiological change measured during the current study was the pulse change. The change in pulse at rest and pulse during effort was measured as an indicator of the degree of change in physical fitness as pulse is greatly dependent on aerobic fitness[20] and also since fitness examinations validated for people with ID[10,21] were not applicable for the current research population due to their limited physical abilities. Pulse during effort and at rest improved significantly. It must be stated that the improvements in pulse at rest and during effort occurred despite the short duration of the intervention period.

The research results indicated a correlation between increased functional ability and improved cardiovascular fitness. Such findings might suggest that one of the elements responsible for low functional performance common among individuals with developmental disabilities is due to the sedentary lifestyle leading to poor physical fitness. Due to the small number of participants, the findings call for further investigation, but should alert professionals to the urgent need for physical intervention programs for this population.

The fact that heart rate at rest deteriorated to almost preintervention values strengthens the findings. The fact that the change was not statistically significant could be explained by the small number of participants (n = 11) and by the fact that children at that age tend to decrease their heart rate consistently.

For years, experts researching the cardiovascular abilities of people with ID have been unified in their warnings of the deficient fitness of this population and identify it as a population at risk for developing vascular and cardiac illness. In addition, researchers have suggested that programs for physical activity of populations at risk for cardiac illnesses should be developed from childhood[15,22]. Deficient fitness was identified as one of the causes of rapid aging and old age illnesses that befall the elderly person with ID at younger ages than those of normal population[14].

The study indicated that children who presented with poor physical fitness at the beginning of the research demonstrated a significant improvement. This fact is reinforced by the fact that when dividing the children to two groups (spontaneously active and inactive), a double improvement was found among the population defined less spontaneously active. Similar findings and identical conclusions were found by Halle et al.[12].

Specific functional subtests such as knee walking, 10-m speed walking, and ascending/descending stairs were found significantly changed, implying a change in pelvic/trunk control achieved by the participants due to the 2-month intervention period. Such a change might suggest that further intervention of the same nature will continue to yield enhancing functional abilities. This assumption requires further investigation.

## CONCLUSIONS

The current study with even a small sample showed that it was possible to construct a training program for improvement of physical fitness operated by unskilled staff and supervised by a physical therapist. Results also showed that such a program improved the physical fitness of children with severe ID and as a result bettered their functional ability, and that both findings were correlated.

The results of the present research offers improved treatment possibilities for the cognitively impaired population. In addition, in contrast to treatment methods examined in the past, which were found to be efficient with populations with moderate cognitive disability as they required the cooperation of the participants, exercise on a treadmill does not require such cooperation and enables even children with severe and profound ID to achieve significant therapeutic results with a low-cost intervention program. It is necessary to continue examining the advantages of the therapeutic approach examined in the present research.

## ACKNOWLEDGMENTS

This study was supported by a grant from the Shalem Foundation. The authors would also like to thank the children, families, and staff members of Beit Issie Shapiro Educational Facility for their cooperation.

## BIOSKETCHES

**Meir Lotan, MScPT,** is a physiotherapist working at the Zvi Quittman Residential Center, The Millie Shime Campus, Elwyn, Jerusalem with special interest in physiotherapy aspects on intellectual disability, Snoezelen, and physical activity for children and adults with intellectual disability. He lectures on assistive technology at the Department of Physical Therapy, Haifa University and Ben Gurion University. E-mail: ml_pt_rs@netvision.net.il

**Eli Isakov, PT, MD,** Professor and Director, Orthopedic Rehabilitation Department and Kinesiology Laboratory, Loewenstein Rehabilitation Hospital, Raanana, Sackler School of Medicine, Tel Aviv University, Israel. E-mail: isakov_e@netvision.net.il

**Shlomo Kessel, MSW,** is the Director of the Sarah Herzog Children Village, Afula in Israel. He has worked for several years as director of residential care centers for persons with intellectual disability and now at a village for children and youth. E-mail: shlomo@emunahafula.org

**Joav Merrick, MD, DMSc,** is Professor of Child Health and Human Development affiliated with the Zusman Child Development Center, Division of Pediatrics and Community Health at the Ben Gurion University, Beer-Sheva, Israel; the Medical Director of the Division for Mental Retardation, Ministry of Social Affairs, Jerusalem; and the Founder and Director of the National Institute of Child Health and Human Development. He has numerous publications in the field of child and human development, rehabilitation, intellectual disability, disability, health, welfare, abuse, advocacy, quality of life, and prevention. Dr. Merrick received the Peter Sabroe Child Award for outstanding work on behalf of Danish Children in 1985 and the International LEGO-Prize ("The Children's Nobel Prize") for an extraordinary contribution towards improvement in child welfare and well being in 1987. E-mail: jmerrick@internet-zahav.net. Website: www.nichd-israel.com
